# First serological evidence of West Nile virus in human rural populations of Gabon

**DOI:** 10.1186/1743-422X-7-132

**Published:** 2010-06-17

**Authors:** Xavier Pourrut, Dieudonné Nkoghé, Janusz Paweska, Eric Leroy

**Affiliations:** 1 Institut de Recherche pour le Développement, UMR 190, Marseille, France; 2 Centre International de Recherches Médicales, BP 769, Franceville, Gabon; 3 Special Pathogens Unit, National Institute for Communicable Diseases, Private Bag X4, Sandringham 2131, South Africa

## Abstract

To investigate West Nile virus (WNV) circulation in rural populations in Gabon, we undertook a large serological survey focusing on human rural populations, using two different ELISA assays. A sample was considered positive when it reacted in both tests. A total of 2320 villagers from 115 villages were interviewed and sampled. Surprisingly, the WNV-specific IgG prevalence was high overall (27.2%) and varied according to the ecosystem: 23.7% in forested regions, 21.8% in savanna, and 64.9% in the lakes region. The WNV-specific IgG prevalence rate was 30% in males and 24.6% in females, and increased with age. Although serological cross-reactions between flaviviruses are likely and may be frequent, these findings strongly suggest that WNV is widespread in Gabon. The difference in WNV prevalence among ecosystems suggests preferential circulation in the lakes region. The linear increase with age suggests continuous exposure of Gabonese populations to WNV. Further investigations are needed to determine the WNV cycle and transmission patterns in Gabon.

## Findings

West Nile virus (WNV) is a mosquito-borne RNA virus belonging to the genus *Flavivirus*, family *Flaviviridae*. Although human WNV infection is generally asymptomatic or causes a 'flu-like illness, life-threatening neurological complications such as meningoencephalitis and flaccid paralysis have been reported [1]. WNV is transmitted in the wild through an enzootic cycle involving birds and ornithophilic mosquitoes [2,3]. WNV has been widely reported thorough the world, including in many African countries [2,4]. However, the distribution of WNV is poorly documented in central Africa. Serological evidence of human exposure to WNV has been reported in the Central African Republic [5], Cameroon [6] and the Democratic Republic of Congo [7], with IgG prevalence rates ranging from 6.6% to 59%. The human WNV IgG prevalence has not been investigated in Gabon. The recent occurrence of a human case with neurological manifestations in Libreville [8] during concomitant chikungunya and dengue outbreaks [9], as well as the detection of specific IgG in horses in some Gabonese cities [10], led us to assess the circulation of WNV in Gabon. We undertook a large serological survey focusing on rural human population and using two ELISA assays: a commercial indirect West Nile virus IgG kit (Panbio diagnostics, Australia) and an ELISA method that has been extensively validated against a serum neutralisation test [11]. We considered as WNV IgG-positive all samples reacting in the two ELISA tests.

**Figure 1:**
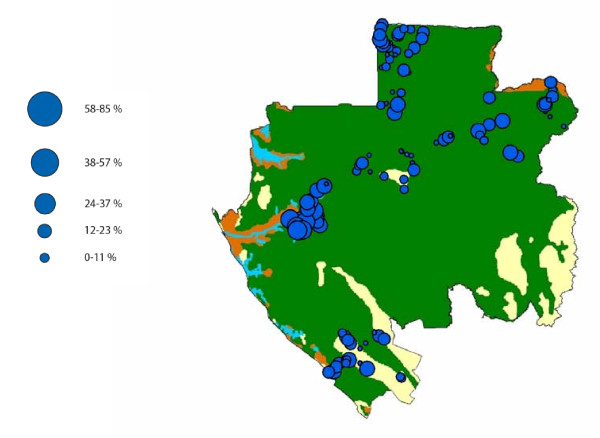
**Location of the Gabonese villages sampled in the forest (green), savanna (yellow) and lakes (brown) regions and corresponding WNV IgG prevalence levels (blue circles)**.

A total of 2320 villagers were interviewed, of whom 1869 (80%) lived in forested regions, 243 (11%) in savannas, and 208 (9%) in the lakes region (Table 1). The WNV-specific IgG prevalence was 27.2% overall (631/2320), 23.7% (443/1869) in the forests, 21.8% (53/243) in the savannas, and 64.9% (135/208) in the lakes region (Figure 1). The villager sample comprised 47.2% of males (1096/2320) and 52.8% of females (1224/2320), and the corresponding WNV IgG prevalence rates were respectively 30% (329/1096) and 24.6% (302/1224). The WNV IgG prevalence rate increased with age from 20.1% (119/591) in the 13-35 year age group to 30.7% (177/576) in the over-60 age group.

**Table 1:** West Nile fever virus-specific IgG prevalence according to villagers' sex and age and the ecosystem.

Characteristics	Variable	Number of Subjects (%)	IgG + (%)	95% CI
All participants		2320 (100)	631 (27.2)	25.4-29.1
Ecosystem				
Forest		1869 (80)	443 (23.7)	21.8-25.7
Savanna		243 (11)	53 (21.8)	16.8-27.5
Lakes		208 (9)	135 (64.9)	58-71.4
Sex	Male	1096 (47.2)	329 (30)	27.3-32.7
	Female	1224 (52.8)	302 (24.6)	22.5-27.1
Age	15-35	591 (25.5)	119 (20.1)	16.9-23.3
	36-50	596 (25.7)	167 (28)	24.4-31.6
	51-60	557 (24)	168 (30.1)	26.4-37.4
	>60	576 (24.8)	177 (30.7)	26.9-34.5

Surprisingly, the overall WNV-specific IgG prevalence was high, at levels similar to those found in countries hit by outbreaks. During the WNV outbreaks that occurred in Sudan in 1898 [12] and in the Democratic Republic of Congo (DRC) in 1988 [7], WNV-specific IgG was found in respectively 59% and 66% of subjects (both IgG and IgM in DRC). During interepidemic periods in Egypt in 1991 [13], Uganda in 1984 [14], and the Central African Republic (CAR) in 1975 [15] and 1979 [16], the WNV-specific IgG prevalence rates were respectively 20%, 16%, 3% and 55%. In countries with no reported clinical cases or WNV isolation, WNV-specific IgG prevalence rates ranged from 0.9% in Kenya in 1987 [17] to 6.6% in Cameroon in 2000 [6] and 27.9% in Ghana in 2008 [18]. The high WNV-specific IgG prevalence detected in this survey suggests that this virus circulates actively in Gabon, despite the lack of reported clinical cases and the low prevalence (about 3%, 2/64) [10] detected in horses sampled in three Gabonese cities. This surprising result may be explained by several factors. Firstly, WNV-specific IgG might arise from aborted WNV infection and antigenic stimulation. Secondly, the lack of reported WNV outbreaks or even isolated cases could be due to the circulation of less virulent WNV strains. Clinical cases may be misdiagnosed and attributed to a similar disease such as malaria or another arbovirosis [19]. Finally, positive serologies might be due to cross-reactions with dengue or yellow fever antibodies, as IgG cross-reactions between flaviviruses have been extensively described in other studies [20,21] and these viruses are known to circulate in several regions of Gabon [9,22].

These preliminary results strongly suggest that WNV circulation is widespread in Gabon. The linear increase in WNV IgG prevalence with age suggests continuous exposure of Gabonese populations to this virus. Moreover, although serological cross-reactions due to non specific WNV antibodies could not been ruled out, the larger difference in the prevalence rates between the different ecosystems suggests preferential WNV circulation in the lakes region. This may be explained by specific ecological features such as mosquito vector species or a higher density of residental and migratory birds [23].

In conclusion, this first serological survey of West Nile virus in Gabonese human populations shows widespread circulation of this virus. Further investigations are needed to determine the WNV cycle and transmission patterns in Gabon.

## Competing interests

The authors declare that they have no competing interests.

## Authors' contributions

Performed the experiment: DN, XP, JP. Analyzed the data: XP, DN, EL. Wrote the paper: XP, EL. All authors read and approved the final manuscript
